# Supplementary material for: Contrasting Effects of Grazing in Shaping the Seasonal Trajectory of Foliar Fungal Endophyte Communities on Two Semiarid Grassland Species
Source: J Fungi (Basel). 2023 Oct 14;9(10):1016. doi: 10.3390/jof9101016 (PMC10608051; doi:10.3390/jof9101016)
Supplement: Supplementary file 1 [file jof-09-01016-s001.zip › jof-2637763-supplementary.pdf]

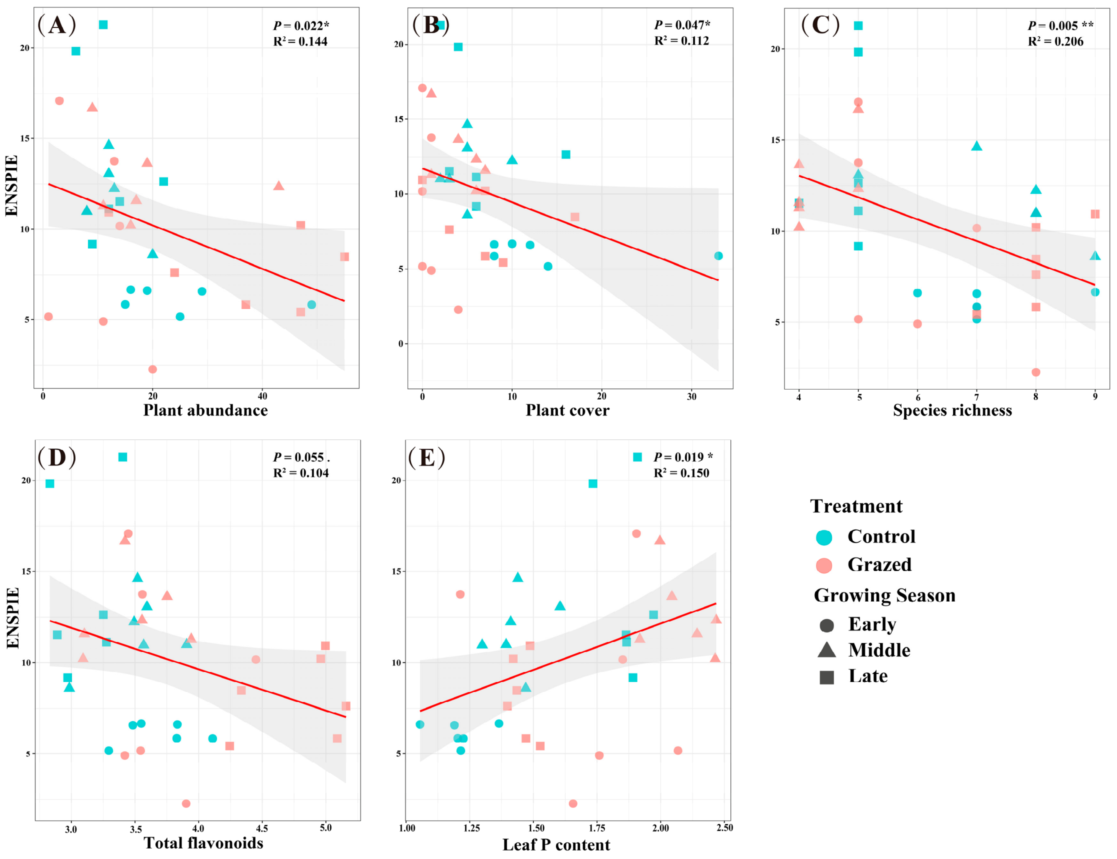

Figure S1. Relationships of the ENSPIE of *Stipa bungeana* with (A) Plant abundance, (B) Plant cover, (C) Species richness, (D) Total flavonoids and (E) Leaf P content (factors significantly correlated with fungal community diversity). The P value and the coefficient of determination (R^2) are shown. Asterisks indicate statistical significance ($P^* < 0.05$; $P^{**} < 0.01$; $P^{***} < 0.001$). Shaded areas represent 95% confidence limits. Grazed is indicated in red, Control is indicated in blue. Early season is marked with a circle, middle season is marked with a triangle and late season is marked with a square.

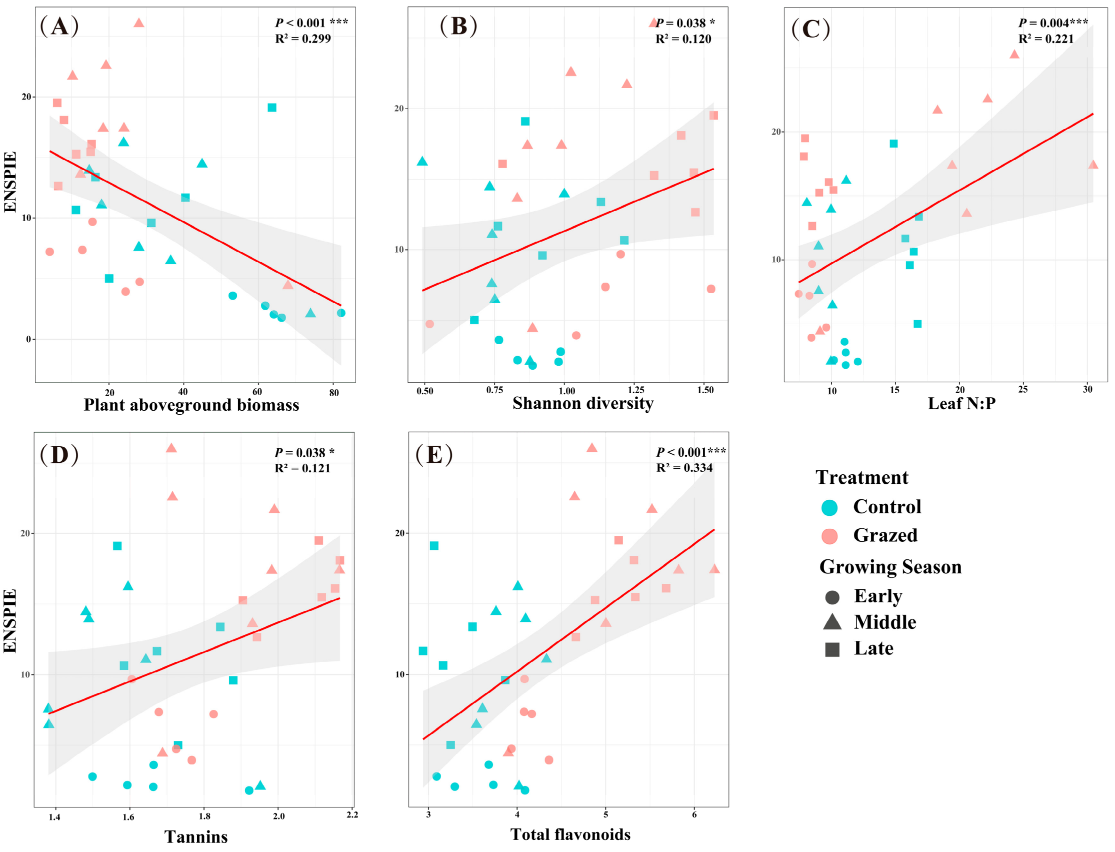

Figure S2. Relationships of the ENSPIE of *Artemisia capillaris* with (A) Plant aboveground biomass, (B) Shannon diversity, (C) Leaf N:P, (D) Tannins and (E) Total flavonoids (factors significantly correlated with fungal community diversity). The *P* value and the coefficient of determination (R^2) are shown. Asterisks indicate statistical significance ($P^* < 0.05$; $P^{**} < 0.01$; $P^{***} < 0.001$). Shaded areas represent 95% confidence limits. Grazed is indicated in red, Control is indicated in blue. Early season is marked with a circle, middle season is marked with a triangle and late season is marked with a square.

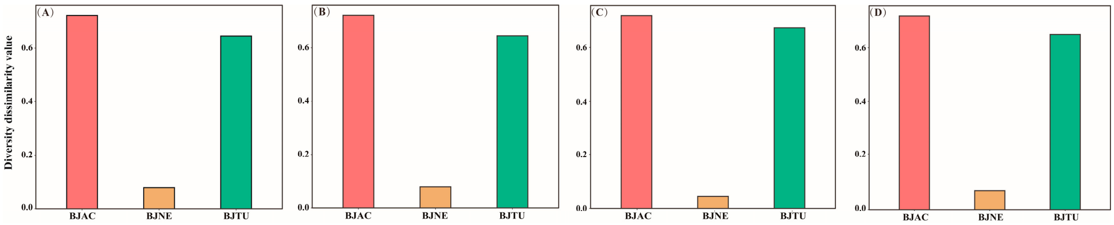

Figure S3. Partitioning of the total β diversity (β_{JAC}, Jaccard dissimilarity index) of *Stipa bungeana* (A-B) and *Artemisia capillaris* (C-D) between (A, C) early and middle season and (B, D) middle and late season into the components of species turnover (β_{JTU}) and change in species numbers (β_{JNE}).

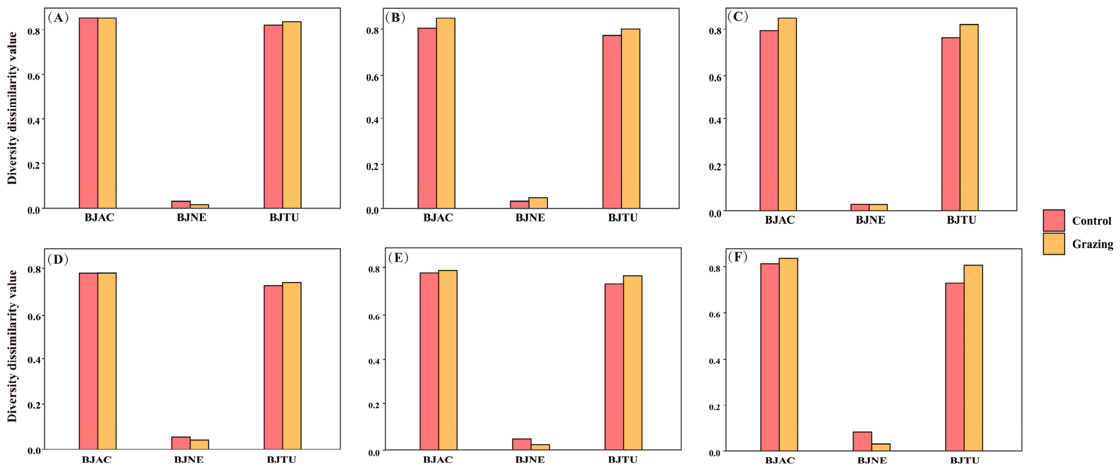

Figure S4. Partitioning of the total β diversity (β_{JAC}, Jaccard dissimilarity index) of *Stipa bungeana* (A-C) and *Artemisia capillaris* (D-F) between the Grazing and Control treatments into the components of species turnover (β_{JTU}) and species loss or gain (β_{JNE}), separately for the (A, D) early, (B, E) middle and (C, F) late season.

Table S1. Summary of the results for grazing treatment (G), growing season (S) and their interactions on the foliar fungal community of *Stipa bungeana* and *Artemisia capillaris* as modelled using linear mixed models for diversity (ENSPIE) and PERMANOVA for community composition (absolute count data). Shown are test statistics and degrees of freedom. *P*-values and R^2-values are reported in Table 1.

Sources	Grazing (G)		Season (S)		G * S	
	x^2/F	*df*	x^2/F	*df*	x^2/F	*df*
Stipa bungeana						
ENSPIE	0.53	1	11.88	2	10.91	2
Coummunity composition	3.03	1	7.03	2	2.35	2
Artemisia capillaris						
ENSPIE	24.20	1	78.89	2	2.82	2
Coummunity composition	8.24	1	19.62	2	5.20	2

Table S2. Effects of grazing treatment, growing season, leaf nutrient content, defensive chemicals and plant community characteristics (factors significantly correlated with fungal community diversity) on the diversity of fungal endophyte communities of *Stipa bungeana* and *Artemisia capillaris.* Parameters in bold are significantly different from zero in the models in which they are included. Importance values are calculated based on the subset of the 128 models that were within 2 AICc units of the best model.

Stipa bungeana	Estimate	z value	*P*	Importance
Intercept	-4652.780	-0.139	0.889	1
MonthJun	3.452	1.930	**0.054**	**0.879**
MonthOct	3.606	1.954	**0.051**	**0.879**
Plant richness	-0.630	-1.147	0.251	0.697
Plant abundance	-0.067	-1.050	0.294	0.654
Plant cover	-0.052	-0.611	0.541	0.362
GrazingUngrazed	0.337	0.467	0.641	0.260
Total flavonoids	<0.001	-0.446	0.655	0.252
Total phosphorus	<0.001	0.112	0.911	0.237
Artemisia capillaris				
Intercept	-3301.630	-0.115	0.909	1
MonthJun	11.802	5.994	**<0.001**	**0.999**
MonthOct	9.509	5.327	**<0.001**	**0.999**
GrazingUngrazed	-4.896	-2.571	**0.010**	**0.947**
Plant Shannon	3.439	1.038	0.299	0.652
Plant biomass	0.025	0.674	0.500	0.420
N:P	0.052	0.589	0.556	0.338
Total flavonoids	<0.001	0.241	0.810	0.198
Tannins	<0.001	-0.208	0.835	0.183

Table S3. Effects of grazing treatment, growing season, plant community characteristics (abundance, cover, aboveground biomass, species richness and Shannon diversity), leaf nutrients (C, N, P, C:N, C:P and N:P) and defensive chemicals (tannins, total flavonoids, total phenols) on composition of fungal endophyte communities in *Stipa bungeana* and *Artemisia capillaris.* Results are from a PERMANOVA using Bray-Curtis distances and 999 permutations of the data.

Stipa bungeana	Df	SumsOfSqs	MeanSqs	F.Model	R^2	*P*
Growing season	2	2.4072	1.20360	7.4743	0.27162	**0.001**
Grazing	1	0.5178	0.51779	3.2155	0.05843	**0.003**
Leaf P content	1	0.2917	0.29169	1.8114	0.03291	**0.045**
Leaf N content	1	0.3562	0.35623	2.2121	0.04020	**0.010**
Leaf C content	1	0.3138	0.31382	1.9488	0.03541	**0.034**
Leaf C:N	1	0.1215	0.12152	0.7546	0.01371	0.743
Leaf C:P	1	0.1286	0.12862	0.7987	0.01451	0.716
Leaf N:P	1	0.2637	0.26374	1.6378	0.02976	**0.045**
Tannins	1	0.1668	0.16682	1.0360	0.01882	0.385
Total flavonoids	1	0.3161	0.31606	1.9627	0.03566	**0.016**
Total phenols	1	0.1854	0.18545	1.1516	0.02093	0.265
Plant abundance	1	0.1921	0.19213	1.1931	0.02168	0.251
Plant cover	1	0.1473	0.14731	0.9148	0.01662	0.558
Plant aboveground biomass	1	0.2483	0.24833	1.5421	0.02802	0.081
Species richness	1	0.1611	0.16113	1.0006	0.01818	0.424
Shannon diversity	1	0.1945	0.19454	1.2081	0.02195	0.262
Growing season * Grazing	2	0.2734	0.13672	0.8490	0.03085	0.686
Residuals	16	2.5765	0.16103		0.29073	
Total	35	8.8623			1.00000	
Artemisia capillaris						
Growing season	2	3.6247	1.81233	19.4356	0.44649	**0.001**

Grazing	1	0.7613	0.76128	8.1641	0.09378	**0.001**
Leaf P content	1	0.3338	0.33375	3.5792	0.04111	**0.004**
Leaf N content	1	0.4991	0.49909	5.3523	0.06148	**0.001**
Leaf C content	1	0.0966	0.09658	1.0357	0.01190	0.376
Leaf C:N	1	0.1342	0.13420	1.4392	0.01653	0.137
Leaf C:P	1	0.0600	0.05996	0.6431	0.00739	0.785
Leaf N:P	1	0.0788	0.07880	0.8451	0.00971	0.552
Tannins	1	0.0802	0.08018	0.8599	0.00988	0.528
Total flavonoids	1	0.1292	0.12918	1.3853	0.01591	0.197
Total phenols	1	0.0995	0.09953	1.0674	0.01226	0.364
Plant abundance	1	0.1005	0.10053	1.0780	0.01238	0.350
Plant cover	1	0.1381	0.13811	1.4811	0.01701	0.138
Plant aboveground biomass	1	0.0709	0.07092	0.7605	0.00874	0.647
Species richness	1	0.1014	0.10142	1.0876	0.01249	0.356
Shannon diversity	1	0.0572	0.05717	0.6131	0.00704	0.804
Growing season * Grazing	2	0.2608	0.13042	1.3986	0.03213	0.158
Residuals	16	1.4920	0.09325		0.18378	
Total	35	8.1182			1.00000	

Table S4. A summary of information on grazed treatment and control treatment.

Treatment	Grazing	Control
Animal species	Sheep	None
Grazing intensity	5.3 sheep/ha	0
Animal number	8 sheep	0
Total area	1.5 ha	1.5 ha
Replicated plots	3	3
Duration	Grazing began in early June with 10 days of grazing and 30 days of rotational interval for a total of three grazing sessions, ending in late September for a total of three months.	None

Table S5. Summary of the results for grazing (G) and growing season (S) and their interactions on the plant community characteristics, leaf nutrient content and defensive chemicals (factors significantly correlated with fungal community diversity) of (A) *Stipa bungeana* and (B) *Artemisia capillaris* as modelled using linear mixed models for diversity (ENSPIE).

Stipa bungeana	Grazing(G)		Season(S)		G*S	
	P	R^2	P	R^2	P	R^2
Plant abundance	0.113	0.040	0.088	0.076	**<0.001**	0.447
Plant cover	**0.008**	0.116	0.308	0.038	**<0.001**	0.374
Species richness	0.215	0.014	0.195	0.029	**<0.001**	0.685
Total flavonoids	**<0.001**	0.213	**0.004**	0.090	**<0.001**	0.717
Leaf P content	**<0.001**	0.152	**<0.001**	0.127	**<0.001**	0.807
Artemisia capillaris						
Log Plant aboveground biomass	**<0.001**	0.318	**<0.001**	0.172	**0.018**	0.536
Shannon diversity	**<0.001**	0.272	**0.033**	0.122	0.434	0.400
Leaf N:P	0.084	0.011	**<0.001**	0.221	**<0.001**	0.872
Tannins	**<0.001**	0.342	**<0.001**	0.142	**<0.001**	0.642
Total flavonoids	**<0.001**	0.553	**<0.001**	0.126	**<0.001**	0.799
